# Exo- and endoscopic two-step approach for recurrent vestibular schwannomas following surgical resection and radiosurgery: How I do it

**DOI:** 10.1007/s00701-025-06625-1

**Published:** 2025-08-05

**Authors:** Kenichiro Iwami, Kazuhito Takeuchi, Yuichi Nagata, Ryuta Saito

**Affiliations:** Department of Neurosurgery, Nagoya University Graduate School of Medicine, 65 Tsurumai-Cho, Showa-Ku, Nagoya, 466-8550 Japan

**Keywords:** Vestibular schwannoma, Recurrence, Exoscope, Endoscope

## Abstract

Intracanalicular residual vestibular schwannoma (VS) after surgical resection is known to cause recurrence. Lesions demonstrating progression despite adjuvant stereotactic radiosurgery necessitate salvage surgery with meticulous tumour resection to prevent recurrence. We describe our exo- and endoscopic two-step approach (EETA), employing an exoscope to remove the extracanalicular component and an endoscope to remove the intracanalicular component under direct visualisation of the fundus. EETA is a viable option for treating recurrent VS, as it enables enhanced visualisation of both extra- and intracanalicular lesions, including the fundus.

**Supplementary Information:**

The online version contains supplementary material available at 10.1007/s00701-025-06625-1.

## Relevant surgical anatomy

The lateral termination of the internal auditory canal (IAC) is marked by the fundus (FIAC; fundus of the internal auditory canal), which forms the medial boundary of the inner ear structure. FIAC is divided into four distinct quadrants: anterosuperior, anteroinferior, posterosuperior, and posteroinferior. The facial nerve is located in the anterosuperior quadrant of the FIAC, known as the facial area. The cochlear nerve occupies the anteroinferior quadrant, referred to as the cochlear area. The superior vestibular nerve is situated in the posterosuperior quadrant, which is called the superior vestibular area. The inferior vestibular nerve is located in the posteroinferior quadrant, known as the inferior vestibular area.

The retrosigmoid approach (RSA), a technique commonly employed by neurosurgeons to excise vestibular schwannomas (VS), facilitates excellent visualisation of the cerebellopontine angle (CPA) and involves opening the posterior wall of the IAC to remove the intracanalicular component. However, the limited visibility of the FIAC remains a key limitation, and the use of endoscopic assistance to improve its visualisation has been reported [1, 7]. Recurrent, residual tumours in the IAC or CPA are often managed with stereotactic radiosurgery [2, 8]; however, re-intervention is required if growth persists [3, 10]. When performing salvage surgery for recurrent VS, a surgical technique that facilitates clear observation of both the IAC and CPA is advantageous. In salvage surgery using RSA, meticulous observation of the FIAC and removal of the intracanalicular component are crucial for enhancing tumour resection and preventing recurrence [2, 9].

## Description of the technique

**Fig. 1:**
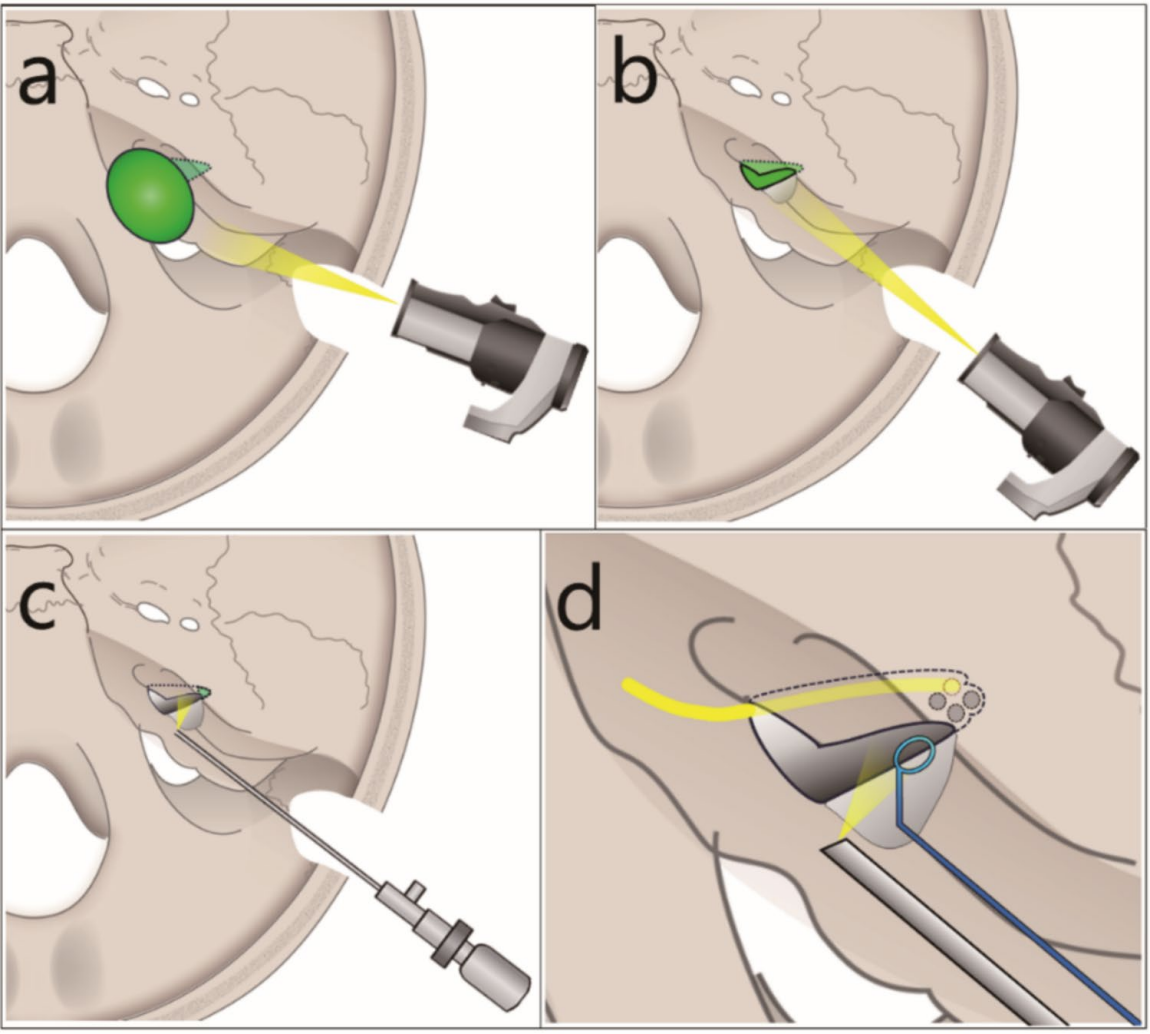
Schematic of the exo- and endoscopic two-step approach (EETA). **a** The first step of EETA: The extracanalicular component is removed using an exoscope. Green, tumour. **b** After widening the IAC opening, if required, the tumour in the medial part of the IAC is also removed under exoscopic visualisation. Green, intracanalicular tumour. **c** The second step of EETA: The residual intracanalicular component (green) is excised using an endoscope. **d** A 70-degree endoscope is utilised to visualise all four quadrants of the fundus (dotted circles). Yellow, facial nerve

The patient underwent surgery in a semilateral position. The RSA is performed through a 2.5 cm bone window by reopening the previous craniotomy site. The exo- and endoscopic two-step approach (EETA) for recurrent VS comprises two steps. First, the extracanalicular component was removed using an exoscope (ORBEYE; Olympus, Tokyo, Japan) using the same technique as that used in microscopic surgery (Fig. 1a). Adhesions and capsule formation resulting from previous treatment can hinder the clear identification of anatomical structures and often necessitate sharp dissection of dense connective tissue. Consequently, salvage surgery requires the use of microsurgical instruments such as scissors under stereoscopic visualisation for precise and safe tumour resection. After widening the IAC opening, if required, intracanalicular tumours extending from the porus of the IAC may also be resected in Step 1, limited to exoscopic visibility (Fig. 1b). When adhesions or scarring render identification of the facial nerve in the CPA challenging, the surgeon may proceed to Step 2 to identify the nerve in the IAC before returning to Step 1.

In the second step, the lateral part of the IAC was examined in detail using rigid endoscopes (0°, 30°, or 70°; outer diameter, 4 mm; Olympus, Tokyo, Japan), and the remanent intracanalicular component was removed (Fig. 1c). If the FIAC was not adequately exposed, we perform further bone removal under endoscopy. Specifically, when removing tumours invading the FIAC, a 70-degree endoscope, which has rarely been used during RSA in previous reports, as employed to visualize all four quadrants of the fundus, thereby minimizing the risk of residual tumour (Fig. 1d) [1, 7]. The use of a curved instrument often obviates the need to remove bone from the FIAC. The EETA procedure for the two representative cases is described below.

### Case 1: Recurrent vestibular schwannoma originating from residual intracanalicular disease. (Figs. 2, 3, and Video 1)

**Fig. 2:**
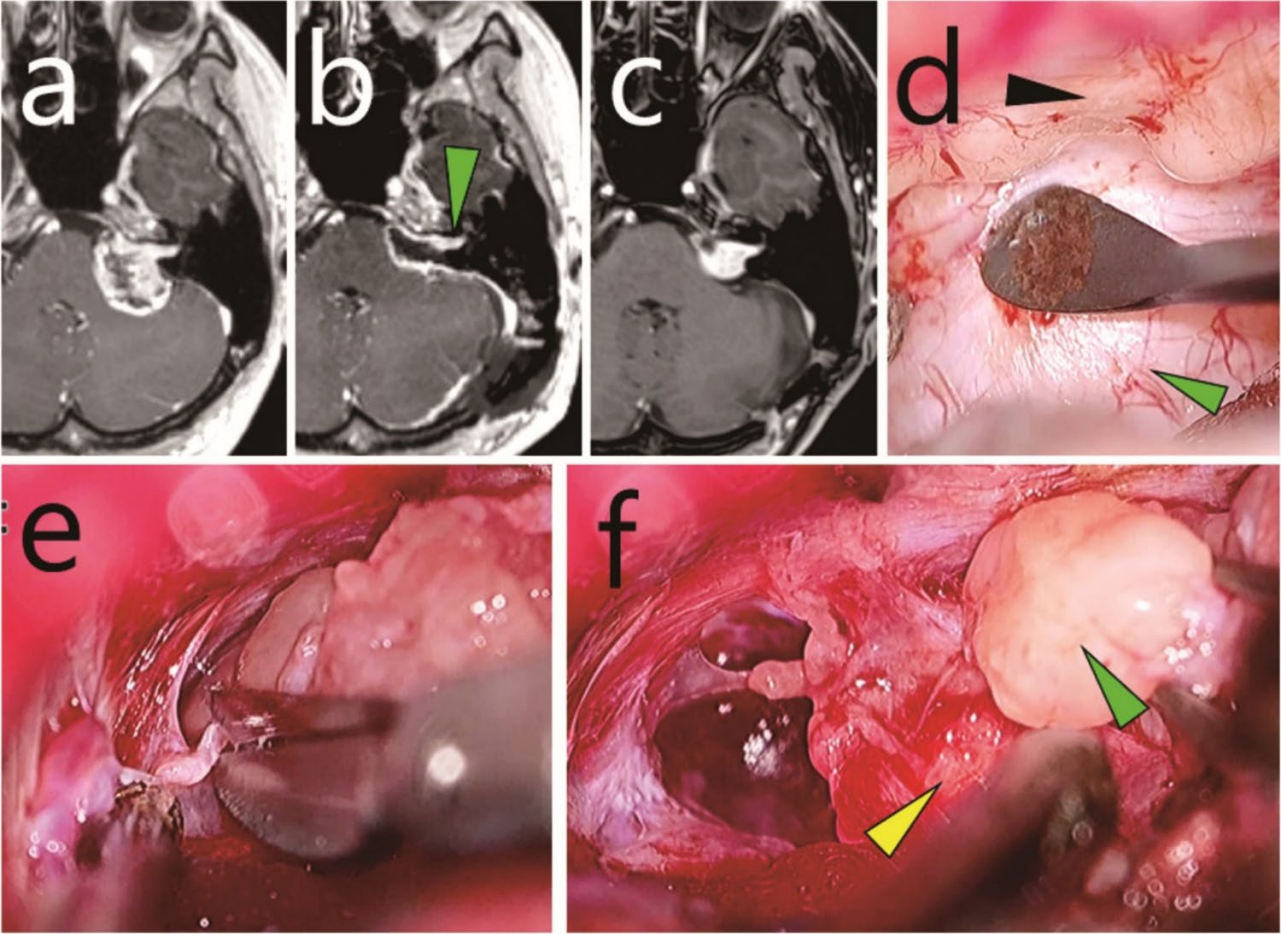
Case 1: Recurrent vestibular schwannoma originating from residual intracanalicular disease. **a** Preoperative gadolinium-enhanced T1-weighted MRI before the initial surgery. **b** Postoperative gadolinium-enhanced T1-weighted MRI after the initial surgery. Green arrowhead, residual tumour within the internal auditory canal (IAC). **c** Gadolinium-enhanced T1-weighted MRI taken 62 months after the initial surgery and 44 months after Gamma Knife radiosurgery. **d-f** Intraoperative exoscopic photographs. **d** Tumour extending from within the IAC to the extracanalicular space (green arrowhead). The black arrowhead indicates the posterior wall of the IAC, partially opened during the previous surgery. **e** Adhesions being divided using scissors. (f) The extracanalicular component (green arrowhead) was dissected from the facial nerve (yellow arrowhead) and removed

**Fig. 3:**
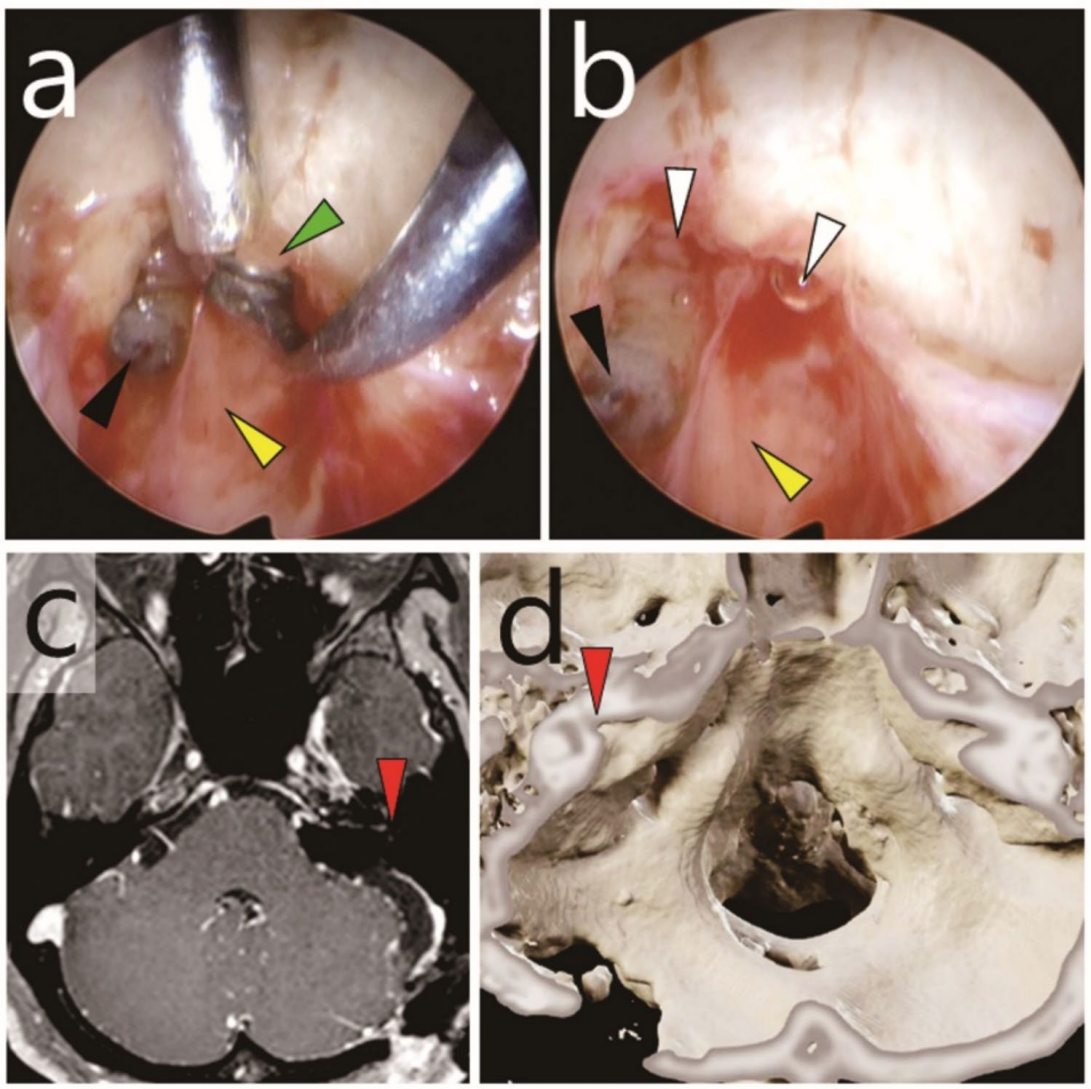
Case 1: Recurrent vestibular schwannoma originating from residual intracanalicular disease. **a**,** b** Intraoperative endoscopic photographs. **a** Resection of the residual tumour (green arrowhead) in the superior vestibular area under 70-degree endoscopic visualisation. Black arrowhead, cochlear area; yellow arrowhead, facial nerve. **b** Observation of the fundus of the internal auditory canal (FIAC) using a 70-degree endoscope. White arrowheads, vestibular areas; black arrowhead, cochlear area; yellow arrowhead, facial nerve. **c** Post-salvage surgery gadolinium-enhanced T1-weighted MRI. Red arrowhead, FIAC (**d**) Post-salvage surgery 3D CT. Red arrowhead, FIAC

One patient presented with left-sided hearing impairment and was found to have left-sided VS (Fig. 2a). Initial surgery resulted in a small residual tumour in the IAC (Fig. 2b). Eighteen months later, the patient underwent Gamma Knife radiosurgery for tumour regrowth (margin dose, 12 Gy; target volume, 1.4 mL). At 62 months post-initial surgery, further growth of the intracanalicular tumour led to referral to our institution (Fig. 2c). The findings included complete hearing loss in the left ear without facial paralysis. Salvage surgery was subsequently performed 68 months after the initial surgery. The patient was discharged one week postoperatively without any complications, including facial paralysis (Fig. 3).

### Case 2: Multiply recurrent vestibular schwannoma (Figs. 4, 5, and Video 2)

**Fig. 4:**
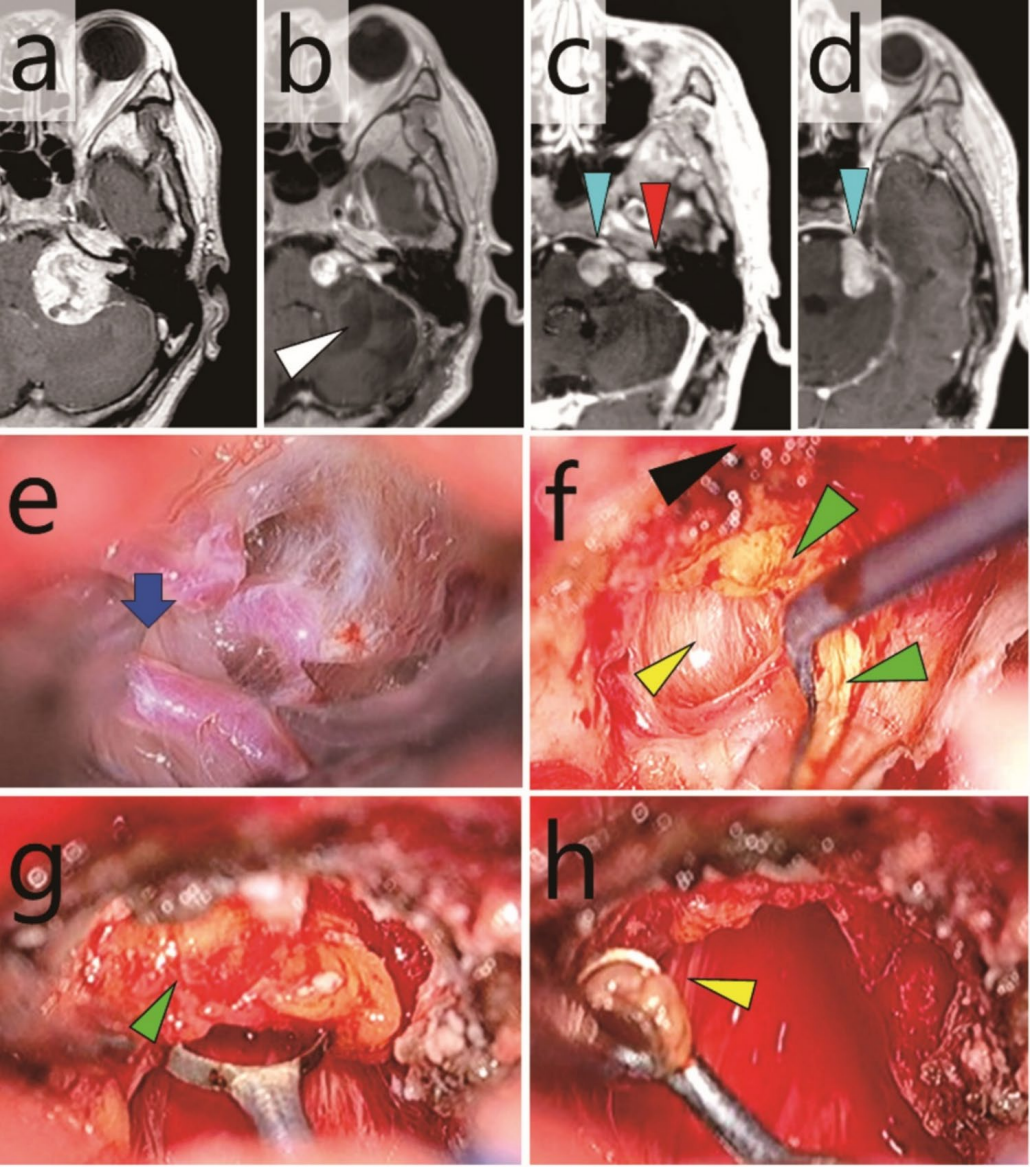
Case 2: Multiply recurrent vestibular schwannoma. **a** Preoperative gadolinium-enhanced T1-weighted MRI before the initial surgery. **b** Gadolinium-enhanced T1-weighted MRI after the first salvage surgery. White arrowhead, cerebellar infarction. **c**,** d** Gadolinium-enhanced T1-weighted MRI taken 60 months after the initial surgery and 25 months after the first salvage surgery. Red arrowhead, intracanalicular tumour; light blue arrowheads, extracanalicular tumour (**e**–**h**). Intraoperative exoscopic photographs. **e** Dense adhesions around lower cranial nerves (blue arrow) and the posterior inferior cerebellar artery. **f** Incision of the capsule and dissection of the intracapsular tumour (green arrowheads) revealed the facial nerve (yellow arrowhead) within the ventral capsule. However, a clear plane between the nerve and capsule could not be established. Black arrowhead, posterior wall of the IAC. **g** The intracanalicular component of the tumour (green arrowhead) was resected. (**h**) The facial nerve (yellow arrowhead) was identified within the IAC

**Fig. 5:**
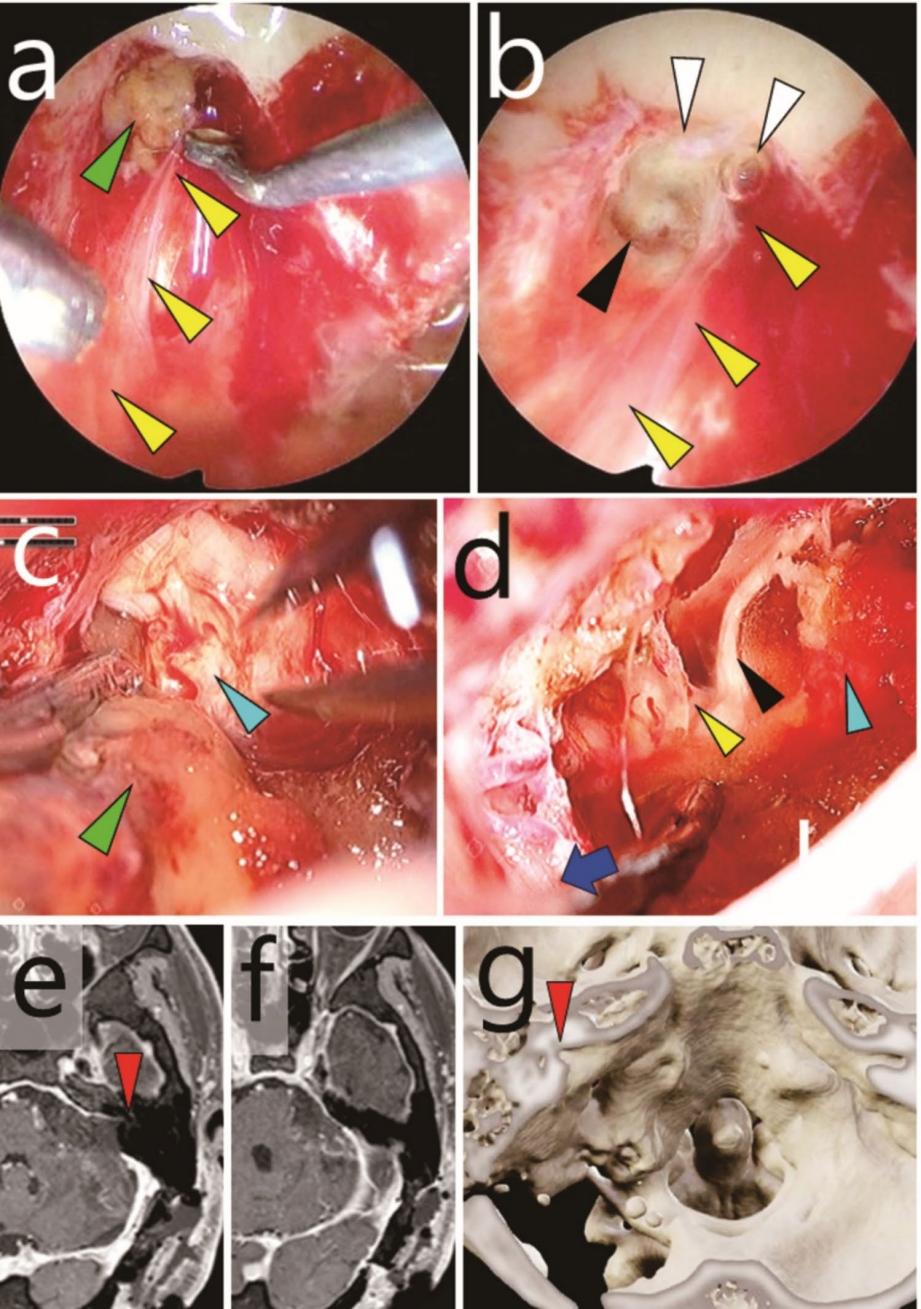
Case 2: Multiply recurrent vestibular schwannoma. **a**,** b** Intraoperative 70-degree endoscopic photographs. **a** Resection of the residual tumour (green arrowhead) in the cochlear area. Yellow arrowheads, facial nerve. **b** Observation of the fundus of the internal auditory canal (FIAC). White arrowheads, vestibular areas; black arrowhead, cochlear area; yellow arrowheads, facial nerve. **c**,** d** Intraoperative exoscopic photographs. **c** After dissection and preservation of the facial nerve, the extracanalicular tumour (green arrowhead) was dissected from the trigeminal nerve (light blue arrowhead). **d** The cerebellopontine angle after tumour resection. Light blue arrowhead, trigeminal nerve covered with oxidised cellulose; black arrowhead, abducens nerve; yellow arrowhead, facial nerve; blue arrow, lower cranial nerves. **e**,** f** Gadolinium-enhanced T1-weighted MRI after the second salvage surgery. Red arrowhead, FIAC (**g**) Post-salvage surgery 3D CT. Red arrowhead, FIAC

One patient presented with left-sided hearing impairment and was found to have left VS (Fig. 4a). Initial surgery resulted in a small residual tumour within and outside the IAC. Thirteen months after the initial surgery, the patient underwent CyberKnife radiosurgery (details are unknown). However, the tumour progressed, leading to referral to another hospital 34 months after the initial surgery. The first salvage surgery was performed 35 months after the initial surgery. Owing to dense adhesions, residual intracanalicular and extracanalicular tumours persisted (Fig. 4b). The patient also experienced cerebellar infarction associated with injury to the left posterior inferior cerebellar artery during this procedure. The residual tumour exhibited indolent growth, and the patient was referred to our hospital 60 months after initial surgery (Fig. 4c, d). Examination revealed complete left-sided hearing loss, along with House-Brackman (HB) grade 3 left-sided facial paralysis. A second salvage surgery was performed at our hospital 64 months after the initial surgery. The patient was discharged one week postoperatively without any complications, including worsening facial paralysis (HB grade 3) (Fig. 5).

**Fig. 6:**
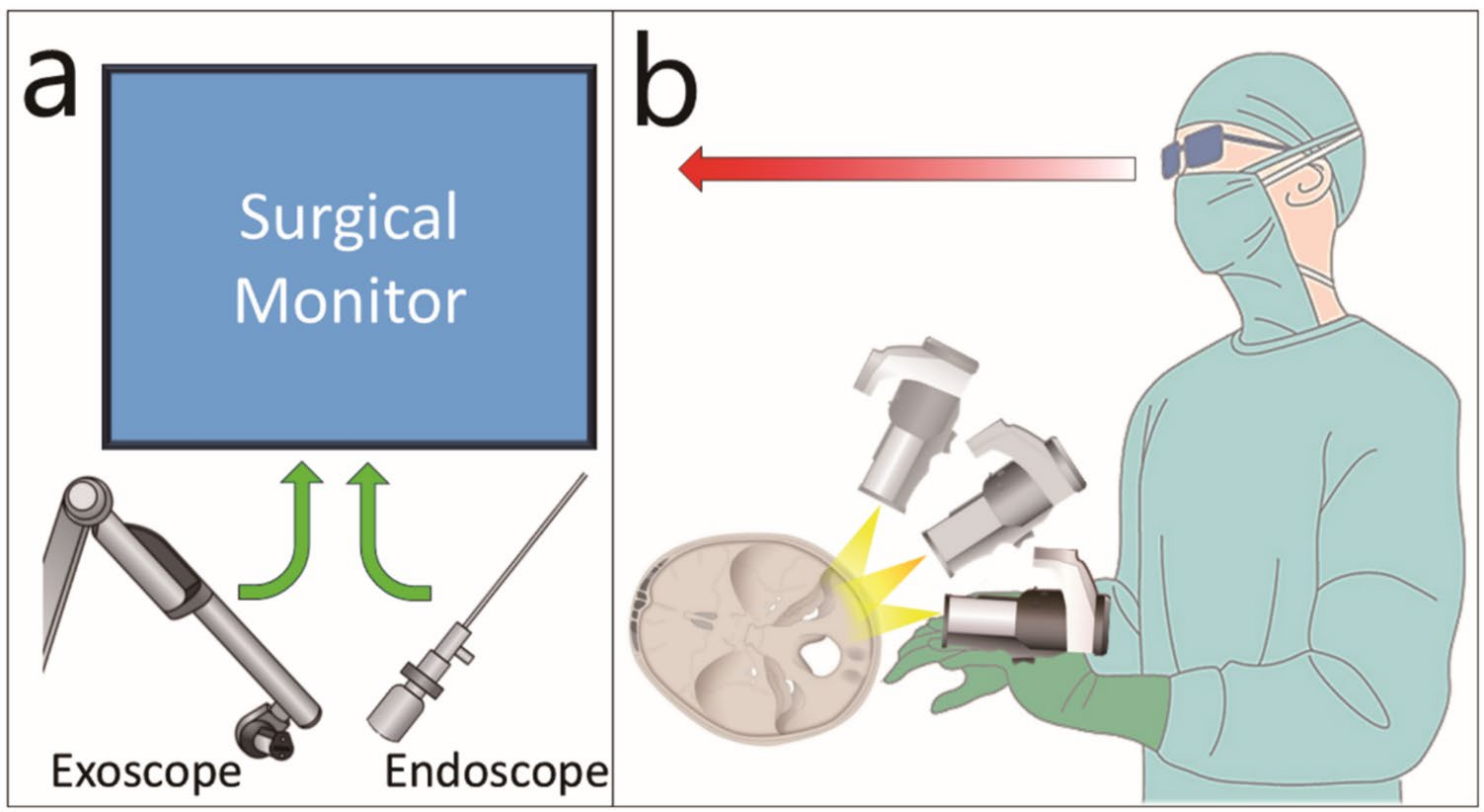
Advantages of using an exoscope (**a**) Both an exoscope and endoscope can display images on a common monitor. **b** Regardless of changes in the exoscope angle, the surgeon can continue the procedure while maintaining a consistent line of sight (red arrow) and posture

An endoscope facilitates superior visualisation of the four quadrants of the FIAC, although the exoscope can be substituted for a microscope. Nevertheless, we recommend the exoscope for several reasons: (1) it enables surgical monitor sharing with endoscopy (Fig. 6a), (2) it allows easy replacement with endoscopy due to its small footprint, (3) it enables three-dimensional image sharing, and (4) it offers superior ergonomics (Fig. 6b) [4, 6]. The combination of the exoscope and the endoscope is particularly suitable for vestibular schwannoma surgery, which requires both delicate dissection under stereoscopic vision and the ability to visualise blind spots.

## Indications

The primary indications include recurrent tumours originating from the residual disease within the IAC, which exhibit both intracanalicular and extracanalicular extensions. For patients who have previously undergone an RSA, salvage surgery can be performed without the need for an additional incision.

## Limitations

VS may exhibit transient enlargement after stereotactic radiosurgery; consequently, decisions regarding salvage surgery must be made cautiously. In cases of complete hearing loss or large tumours, the translabyrinthine approach should also be considered, as it avoids cerebellar retraction and facilitates early identification of the facial nerve within the IAC.

## How to avoid complications

Identification of the vasculature of the cerebellopontine angle, including petrosal vein, anterior inferior cerebellar artery, and posterior inferior cerebellar artery, can be challenging due to scarring and adhesions; therefore, preoperative assessment is essential. Angiography is considered superior; however, evaluation can also be achieved with 3D CT. We aimed for delicate manipulation to preserve the neurovascular structures. In particular, regarding the facial nerve, highly meticulous dissection is required. In cases of strong adhesions, excision should be limited to the extent necessary to preserve the nerve. For non-progressive residual tumours, preservation of normal anatomy should be prioritised, with resection considered only within the constraints of this priority [9, 10].

## Specific information for the patient

General surgical risks, such as infection and haemorrhage, may also be observed. The risk of neurovascular injury during the resection of a recurrent tumour is relatively high compared to the initial surgery, potentially due to previous treatments that may have caused strong tumour adherence to the brainstem and surrounding neurovascular structures.

## Ten key-point summary

1. EETA, a surgical procedure employing both exoscopic and endoscopic visualisation, is used as salvage surgery for recurrent VS.
2. The primary indications for EETA include recurrent tumours originating from the residual disease within the IAC.
3. The RSA is performed through a 2.5 cm bone window created after reopening of the prior craniotomy site.
4. In the initial step, tumours extending extracanalicularly and those located medially within the IAC are excised under exoscopic visualisation.
5. Adhesions and capsule formation can obscure anatomical structures, often necessitating sharp dissection of dense connective tissue.
6. In the second step, the residual tumour within the IAC is excised using an endoscope.
7. A 70-degree endoscope is used to achieve clear visualisation of all four quadrants of the fundus when removing tumours invading the FIAC.
8. If facial nerve identification in the CPA is difficult due to adhesions or scarring, Step 2 may be performed first to identify the nerve in the IAC before proceeding with Step 1.
9. Because salvage cases often have dense adhesions, careful micro-dissection is required; the combined exo-endoscopic view facilitates but does not eliminate risk.
10. The advantages of combining an exoscope and an endoscope include the ability to use a shared monitor, ease of instrument switching, and ergonomic benefits.

## Supplementary Information

Below is the link to the electronic supplementary material.

ESM 1 Supplementary Material 1 (MP4 116 MB)

ESM 2 Supplementary Material 2 (MP4 134 MB)

## Data Availability

No datasets were generated or analysed during the current study.

## Abbreviations

VS: Vestibular schwannoma
EETA: Exo- and endoscopic two-step approach
IAC: Internal auditory canal
FIAC: Fundus of the internal auditory canal
RSA: Retrosigmoid approach
CPA: Cerebellopontine angle
MRI: Magnetic resonance imaging
